# Impact of social support and mindfulness in the associations between perceived risk of COVID-19 acquisition and pregnancy outcomes in Iranian population: a longitudinal cohort study

**DOI:** 10.1186/s40359-023-01371-4

**Published:** 2023-10-11

**Authors:** Zahra Sharifi-Heris, Leila Amiri-Farahani, Zahra Shahabadi, Mohaddeseh Sanaei

**Affiliations:** 1 Sue & Bill Gross School of Nursing, University of California at Irvine, Irvine, CA USA; 2 Department of Reproductive Health and Midwifery, Nursing and Midwifery Care Research Center, School of Nursing and Midwifery, Iran University of Medical Sciences, Tehran, Iran; 3 Student Research Committee, Department of Reproductive Health and Midwifery, School of Nursing and Midwifery, Iran University of Medical Sciences, Tehran, Iran

**Keywords:** Mindfulness, “COVID-19 risk”, Pregnancy outcomes, Social support, Neonatal outcomes, Maternal outcomes

## Abstract

**Background and aims:**

Various devastating infection outbreaks including COVID-19, threat both mother and fetus health. These life-threating outbreaks as potential harms are highly associated with relevant perceived risk. Social support and mindfulness are two factors that may moderate the associations between the perceived risk of COVID-19 and pregnancy outcomes. In this study we investigated the potential moderating impact of social support and mindfulness in the aforementioned association.

**Methods:**

This study is a longitudinal cohort study in which 483 Iranian pregnant women in Tehran have been studied. Perceived risk of COVID-19 questions, Mindful Attention Awareness Scale (MAAS), and Multidimensional Scale of Perceived Social Support (MSPSS) were used through an online platform to assess the independent variables during pregnancy. Neonatal and maternal outcomes including gestational diabetes, gestational hypertension, preeclampsia, abortion, birth weight, and gestational age at birth, was extracted from Electronic Health Record (EHR) after childbirth as the dependent variables. The aim of the study is to investigate whether social support and mindfulness can affect the associations between perceived risk of Covid-19 acquisition and pregnancy outcomes.

**Results:**

Perceived risk of COVID-19 was negatively associated with pregnancy outcomes including birth weight (-28, 95% CI [-53, -3.4], *p* < .05) and gestational age at birth (-0.9, 95% CI [-2,0.11], *p* < .05). However, social support could not moderate these associations. Mindfulness, on the other hand, moderated the association between perceived risk and stillbirth meaning that by increasing mindfulness, the association between the perceived risk and stillbirth may also be increased (OR = 0.03; *p* < .05).

**Conclusion:**

The findings of this study showed that social support lacks the moderating impact on the association between perceived risk of COVID-19 and pregnancy outcomes. Mindfulness, on the other hand, indicate a positive moderating impact for the association between perceived risk of Covid-19 and stillbirth. More studies in different populations are suggested to investigate the impact of mindfulness and social support on the association between perceived risk and pregnancy outcomes.

## Introduction

Risk is pervasive fact that affects the individuals these days. Growing changes caused by natural disaster, diseases outbreak, technological advances, individualization, modified nutrition products, and subsequent environmental adaptions complicate the risk management for both caregivers and caretakers [1]. The perceived risk during pregnancy and accompanied concerns may threaten both pregnant women and unborn babies’ lives by affecting morbidity and mortality [2]. According to literature, there are two basic groupings of potential harms during pregnancy: harms to the infant [3]; and potential harms to the mother [4]. The perceived risk toward aforementioned harms can be intensified when a woman is exposed to extra harm and risk sources including specific environmental crisis for which potential maternal and neonatal damages can be ranged from unknown to severe [5].

Emerging infectious diseases may be perceived as potential harms and risks for public physical and mental health including pregnant women across the world. The history of devastating outbreaks with different geographical origins including Ebola, West Nile encephalitis, severe acute respiratory syndrome (SARS), avian flu, and recently COVID-19 indicate the global effect of the infectious epidemics [6]. The COVID-19 is a worldwide disaster reported to have higher reproductive rate than what WHO estimated for that [7]. The mortality rate is high in some developed and developing countries including Iran [8]. Along with unavoidable fatal impact of this pandemic, the damaging indirect effects including quarantine, supply deficiency, information lack, and uncertainty in potential damages influence people’s lives [9]. Pregnant women are not exceptions for these consequences. Such widespread disasters have potentiality to affect the maternal mental health and perceived maternal risk and consequently pregnancy outcomes [10, 11]. Several studies during covid-19 pandemic indicated the high perceived risk toward Covid-19 infection in pregnant women [5, 12–14]. However, none of them assessed if the increased risk has potential to affect pregnancy outcomes. Studies that concerned with pregnancy outcomes, often considered the direct impact of Covid-19 viral infection and consequent pregnancy outcomes in the infected pregnant individuals [15]. There is a study that compared the birth outcomes (e.g., preterm birth, stillbirth, abortion, hypertensive disorders such as preeclampsia) before and during the Covid-19 pandemic [16]. The findings of this study showed significant increases in pregnancy-related complications and maternal death during pandemic as compared to pre pandemic period. Although part of this increase is related to the direct impact of infection, there should be explanation on the increase in uninfected pregnant women. This explanation can pass through the perceived risk of Covid-19 acquisition.

Additionally, in this scenario, there are known protective factors that may improve health outcomes [17]. Social support is one of the important factors playing complex role on women’s well-being. Social support may affect maternal and neonatal well-being including mental and physical health [18–20]. Evidence was found for main, moderating effects of social support on women’s well-being under challenges such as physical and mental abuse and stress [21]. However, literature lack its potential impact on the perceived risk related to COVID-19 pandemic. Social support has been defined as “support accessible to an individual through social ties to other individuals, groups, and the larger community” [22]. It is worth mentioning that the degree of need for social support may depend on the developmental and existing psychophysical stages of the person who is receiving the support. For example, a pregnant woman, who experiences various physical and psychological alterations, has more in need of social support from family and friends to overcome the relevant challenges [23]. This need is expected to be intensified when dealing with COVID-related issues including catching the virus and its negative impact on the unborn baby. Given to literature that supports its protective impact, it seems that social support has potential to affect the association between the perceived risk of COVID-19 and pregnancy outcomes.

Over the past decades, also, a new component named “mindfulness” is widely accepted to influence risk perception [24], health outcomes [25], pregnancy outcomes [26, 27], and high quality of life [28]. Its impact during COVID-19 pandemic and accompanied perceived risk, however, is not known. Mindfulness is intrinsically an adaptive mental state, often described as the attention to moment-to-moment experience with an accepting and nonjudgmental attitude [29], or as a receptive attentiveness to present experience that can improve both mental and physical health [30, 31]. Even though, emerging studies have no consensus regarding the nature of mindfulness component, there is a tendency that mindfulness is highly associated with self-consciousness [32]. Increased self-consciousness may either mitigate or intensify one’s perception toward the existing risk. Understanding its potential mechanism of action during COVID pandemic may help to identify potential coping resources in similar environmental pandemics.

It is important to identify concepts that may affect pregnant women in overcoming the immediate and residual effects of COVID pandemic. Despite its importance, literature provides no adequate understanding of the aforementioned concepts. Studies often investigated the mental health impact of pandemics and specifically anxiety and perceived risk [10, 33]. We still lack knowledge on whether this mental distress can affect pregnancy outcomes and how it can be moderated by known protective factors including social support and mindfulness. The current study aimed to examine the association between perceived risk of COVID-19 and maternal-neonatal outcomes affected by social support and mindfulness during COVID-19 pandemic.

## Material and method

This is a longitudinal cohort study in which exposures (perceived risk of Covid-19), mediators (social support, and mindfulness) were measured during pregnancy, and outcomes (neonatal and maternal outcomes) were measured after childbirth when the information were documented in the patient medical records.

### Sampling

**Fig. 1:**
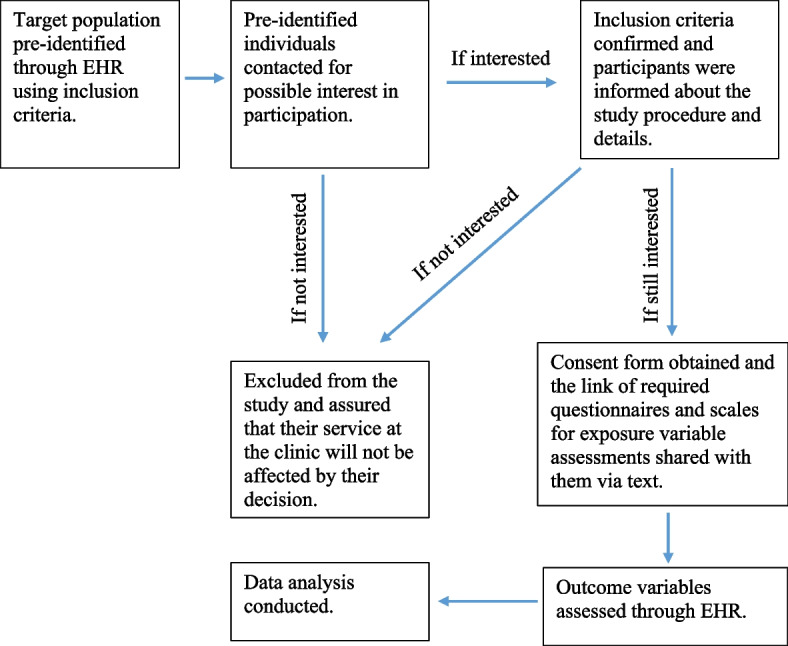
Sampling Strategy of the study

After obtaining institutional review board approval and informed consent, individuals who met the entry criteria were recruited into the study using non-probability purposive sampling from the list of all pregnant individuals in the EHR system and proceeded to the assessment step (Fig. 1). The recruitment started in Jan 2021 and completed in June 2021 for about 6 months. Inclusion criteria included: Healthy-identified pregnancy, no current or previous exposure to the Covid-19, access to internet and smart phone, ability to speak Farsi. To improve internal validity when exploring the connection between exposure and outcome variables, the study concentrates on healthy pregnant individuals as the target group. Inclusion of high-risk women may introduce unrelated variables, potentially compromising result validity. Moreover, high-risk pregnancies inherently induce mental distress, making it challenging to isolate pandemic-related risk perception from pre-existing stress. Given that the majority of pregnancies are deemed healthy, focusing on this predominant group and ensuring their well-being seems to be essential during pandemic. American Congress of Obstetricians and Gynecologists [ACOG] criteria applied for identification of healthy pregnant individuals [34]. The accessible populations were the pregnant women who had Electronic Health Record (EHR) in the clinics that are affiliated by Iran University of Medical Sciences. Healthy pregnant women were pre-identified through EHR from the list of all pregnant individuals whom account showed no restriction for research participation, and contacted via phone if they were interested in participation. If interested, inclusion criteria such as ability to read and write in Farsi language, and accessibility to internet for online questionnaire, were assessed. No additional inclusion criteria considered. All participants were informed about the aim and study protocol and signed an online written informed consent.

### Sample size

The G-power software version 3.1.9.6 was used for the statistical power of the study. Considering the previous relevant study in the SARS pandemic [10], we applied a = 0.05, power = 0.8, proportion p1 = 0.4, and p2 = 0.2. The sample size was calculated to be 246 for the original study. However, since the number of outcomes was twice more in our study, we aimed for 492 subjects. Considering 22% attrition rate [35] we aimed for 636 sample size.

### Measures

The exposure, moderating, and outcome variables were assessed at recruitment through an online platform name Porsline (survey.porsline.com).

#### Exposure(s)

Perceived risk of COVID-19 as the exposure variable, we used two questions applied in a relevant study [36].

#### Potential moderator(s)

Mindfulness and Social support as potential mediators were assessed using Mindful Attention Awareness Scale (MAAS) [37], and Multidimensional Scale of Perceived Social Support (MSPSS) [38], respectively. The MAAS with Cronbach’s α = 0.88–0.89 [39, 40] and MSPSS with Cronbach’s α = 0.83 [41] are valid scales used in Iranian population. The possible lowest and highest score for social support (lowest: 12, highest: 84) and mindfulness (lowest: 15, highest: 90) is certain.

#### Outcomes

Neonatal and maternal outcomes including gestational diabetes (GDM), gestational hypertension (HTN), preeclampsia, abortion, birth weight, and gestational age at birth, was extracted from Electronic Health Record (EHR) after childbirth.

#### Covariates

Gestational age, maternal age, gravidity, Body Mass Index, educational years, income, and job were considered as the potential confounding factors. Except for income and job that were self-reported, other covariates were assessed in the electronic health record (EHR).

### Statistical analysis

R software version 2022.02.3 has been used for statistical analysis. Linear and logistic regression have been applied for adjusted (multivariate) and unadjusted (bivariate) models. For unadjusted model, we ran the model for all candidate independent variables (outcome ~ exposure OR covariates). For adjusted model, we inserted all covariates regardless of their *p*-value and ran the model (outcome ~ primary exposure + covariates).

Moderation analysis was performed for those that indicated significant associations between the main outcome and exposure in regression analysis. Interaction term applied to run the moderation analysis (outcome ~ primary exposure*potential moderator). The *p*-value ≤ 0.05 is considered significant for confidence interval of 95%.

## Results

**Fig. 2:**
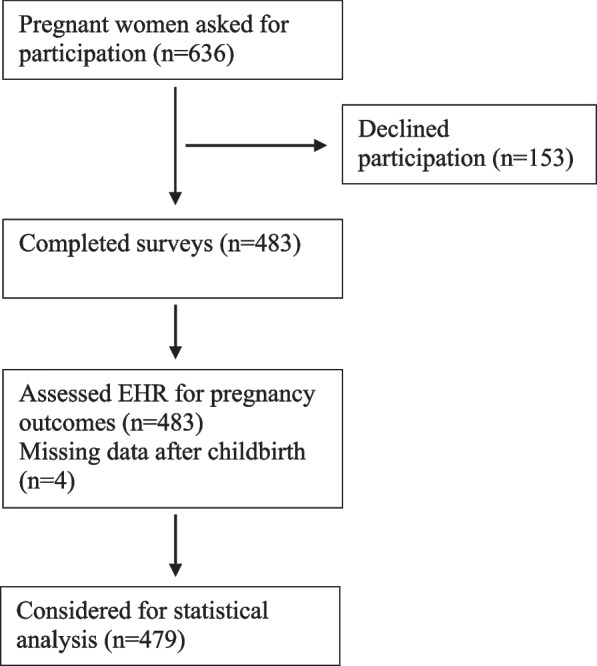
Flow chart of the study

A total of 483 women were returned a completed questionnaire for a response rate of 76% (completed/ recruited) and an average completion time of 15 min. Four women were missing data for pregnancy outcomes through EHR. Due to the small size of the missing data, deletion method was applied to manage the missing data. Finally, the data for 479 women considered for statistical analysis. The flow of the recruitment of participants and data analysis is specified and reported (Fig. 2).

The average duration between the exposure-assessment and outcome-assessment was nine weeks. The covariates were selected based on their potential impact on the pregnancy outcomes in the existing evidence. For example, primiparity and old age are two significant predictors of adverse pregnancy outcomes [42]. Maternal obesity also has been linked to the poor pregnancy outcomes in the literature [43]. Additionally, poor sociodemographic situation in the studies were associated with maternal complications [44]. As demonstrated in Table 1 (descriptive characteristics), women were 30 years old on average with 4 years of education history, starting from primary school. About half of the women were multigravida and gestational age was 27 weeks among the participants. All were married. About 83% had currently no job and 84% had monthly income less or equal to five million Toman. None of the participant were drug, alcohol or cigarette consumers and had no adverse medical or obstetric history. Given to the possible highest score for social support (Mean [SD]: 66.75 [12.66]), it seems that pregnant women experienced more than average social support and less than average mindfulness (Mean [SD]: 30 [9.04]) during COVID-19 pandemic. However, there is no cutoff to interpret this score in a valid way.

**Table 1:** Descriptive analysis of the participants (*n* = 479)

Characteristics		Statistics	Range
**Gestational age** (mean [SD])		27.9 (8.6)	[13,32]
**Maternal age in years** (mean [SD])		30.27 (6.12)	[18,35]
**BMI** (mean [SD])		25.8 (1.25)	[21.9, 24.5]
**Gravidity** (n [%])	Primigravida	246 (51%)	
	Multigravida	243 (49%)	
**Educational years** (mean [SD])		4.25 (0.95)	[12,23]
**Income** (n [%])	Less than 5 million Toman	404 (84%)	
	Greater than 5 million Toman	75 (16%)	
**Job** (n [%])	Have jobs	79 (17%)	
	Have no jobs	400 (83%)	
**Social support** (mean [SD])		66.75 (12.66)	[18,84]
**Mindfulness** (mean [SD])		30 (9.04)	[17,72]
**Perceived risk of COVID-19** (mean [SD])		6.3 (1.7)	[2,10]

In unadjusted analysis, we could not find significant associations between perceived risk of COVID-19 and pregnancy outcomes except for gestational age at birth and birth weight. Interestingly, these two outcomes maintained their negative significant associations with perceived risk when adjusted to socio-demographic factors such as age, gestational age, gravida, education, job, and income. It seems that by increasing in perceived risk of COVID-19, gestational age and birth weight may be decreased.

Among potential confounding factors, gravida showed significant positive impact on GDM (1.04; *p*-value = 0.006; 95% CI [1.1, 1.8]). This means that by increasing in gravida, the odd ratio for GDM may be also increased. Educational years positively affected birth weight in adjusted model (0.2; *p*-value = 0.02; 95% CI [0.11, 0.97]). However, this impact did not affect the significance of the association between the perceived risk of COVID-19 and birth weight in the adjusted model compared with the unadjusted model (*p* = 0.04 in unadjusted vs. *p* = 0.02 in adjusted model) (Table 2).

**Table 2:** Adjusted and unadjusted model for the association between perceived risk of COVID-19 and pregnancy outcomes

Pregnancy outcomes	Measure of association	Unadjusted	Adjusted
GDM	OR (CI)	-0.11(-0.26,0.03)	0.89 (0.76,1.05)
HTN	OR (CI)	0.13 (-0.05,0.33)	1.13 (0.93,1.39)
Preeclampsia	OR (CI)	0.06 (-0.33,0.49)	1.09 (0.71, 1.73)
Abortion	OR (CI)	-0.5 (-1.15,0.11)	0.59(0.29,1.15)
Stillbirth	OR (CI)	-0.15 (-0.53,0.25)	0.83(0.55,1.28)
Gestational age at birth	Β (CI)	-1.04*(-2.05, 0.03)	-0.9(-2,0.11) *
Birth weight	Β (CI)	-24 *(-49, -0.73)	-28(-53, -3.4) *

*OR* Odd ratio, *B* Beta, *CI* Confidence interval

^#^ adjusted to maternal age, gestational age, BMI, gravida, education, job, and income

^*^P-value < .05

In the moderation analysis, social support did not indicate any moderation impact on the associations between perceived risk of COVID-19 and the pregnancy outcomes. Mindfulness, however, demonstrated significant role in moderating the association between perceived risk of COVID-19 and still birth (*p* = 0.04). Interpreting this role, by increasing mindfulness score, the odd ratio of still birth may be increased (Tables 3 and 4).

**Table 3:** Moderation analysis of social support

Pregnancy outcomes	Risk*Social support (ITC[CI])	*P*-value	Risk	Social support
**GDM**	-0.006 (-0.01,0.005)	0.28	0.34 (-0.51,1.22)	0.05 (-0.02,0.13)
**HTN**	0.002 (-0.04,0.01)	0.8	-0.01 (-1.15,1.17)	0.01 (-0.09,0.12)
**Preeclampsia**	-0.01 (-0.04,0.01)	0.41	0.98 (-1.3, 1.05)	0.09 (-0.12, 0.29)
**Abortion**	0.002 (-0.04,0.05)	0.92	-0.69 (-4.49, 2.7)	0.02 (-0.21, 0.28)
**Stillbirth**	0.009 (-0.02,0.03)	0.5	-0.79 (-2.8,1.49)	-0.03 (-0.2,0.16)
**Gestational age at birth**	-0.001 (-0.12.0.08)	0.97	1.17 (-3.69,11.62)	0.01 (-0.31, 0.45)
**Birth weight**	-0.002 (-0.1,0.1)	0.98	0.29 (-9.13,8.89)	-0.1 (-1.01,0.36)

*ITC* Interaction term coefficient

Interaction term applied (outcome ~ COVID Risk*potential moderator)

^*^P-value < 0.05

**Table 4:** Moderation analysis of mindfulness

Pregnancy outcomes	Risk*Mindfulness (ITC [CI])	*P*-value	Risk	Mindfulness
GDM	0.001 (-0.01,0.01)	0.81	-0.14 (-0.69,0.39)	-0.01 (-0.12,0.09)
HTN	0.002 (-0.02,0.02)	0.84	0.08 (-0.63,0.82)	-0.04 (-0.22,0.11)
Preeclampsia	0.01 (-0.03,0.05)	0.6	-0.29 (-1.54,1.14)	-0.04 (-0.32, 0.22)
Abortion	-0.02 (-0.07,0.02)	0.38	0.36 (-1.67,2.4)	0.19 (-0.08,0.49)
Stillbirth	0.03* (-0.005,0.06)	0.04	-1.16 (-2.22, 0.08)	-0.19 (-0.45, 0.05)
Gestational age at birth	0.001 (-0.09,0.1)	0.98	1.02 (-2.9,6.55)	-0.08 (-0.49,0.31)
Birth weight	0.003 (-0.09,0.09)	0.94	-0.002 (-3.25,3.11)	-0.04 (-0.57,0.71)

*ITC* Interaction term coefficient

Interaction term applied (outcome ~ COVID Risk*potential moderator)

^*^P-value < 0.05

## Discussion

Our study results indicated that perceived COVID-19 risk may negatively affect pregnancy neonatal outcomes such as birth weight and gestational age at birth but not the maternal adverse outcomes. In the moderation analysis, social support did not indicate any significant role in moderating the association between the exposure and outcome variables. Mindfulness, on the other hand moderated the association between the perceived risk of Covid-19 and stillbirth. Although literature lacks information on the impact of the COVID-19 risk perception on birth weight and gestational age at birth, studies support the association between maternal stress and low birth weight and preterm birth [45, 46]. Studies often have concentrated on direct impact of COVID-19 infection and have ignored the mental impact of COVID-19 pandemic on the pregnancy outcomes. For example, Wei et al. (2021) systematically investigated the pregnancy outcomes in infected pregnant women and concluded that direct exposure with COVID-19 virus can lead to preeclampsia, preterm birth, and stillbirth [15]. In another study by Wilkinson et al. (2022), it is indicated that iatrogenic preterm birth was more common in COVID-19-infected individuals than controls. Although understanding the pathological impact of COVID-19 virus is crucial, it is timely to explore more social and mental aspect of pandemic in inducing poor pregnancy outcomes [47].

In this study, we tried to cover this critical gap by considering the risk perception and mental impact of the COVID-19 pandemic. In our study, the indicated positive link of COVID-19 perceived risk with low birth weight and preterm birth is justifiable through the possible mental distress due to concerns related to COVID-19 acquisition during the pandemic. In this association, adjusting the potential confounding factors such as income, maternal age, gestational age, and joblessness did not affect the association between the main exposure and outcome variables. However, educational years and gravida indicated significant impact on birth weight and GDM, respectively. Existing literature support the impact of multi gravidity on GDM [48, 49]. Studies also support the positive impact of maternal education on birth weight [50, 51]. Interestingly, joblessness and low income, which are the expected consequences of the pandemic, did not affect the pregnancy outcomes. This conflicts with the studies that suggested poverty as a strong risk factor for poor pregnancy outcomes [52, 53]. This is explainable by the long-lasting economic difficulties in Iran that may have led to high resilience in long term. This resilience may be gained through eventual adaptation in response to the commonly occurred stressor (poverty in this case) [54].

In this study, we included pregnant women who were healthy according to the ACOG criteria. This was to control for known risk factors that may affect the pregnancy outcomes. This increases the internal validity of the study and provides more reliable results to understand causal relationship. However, we accept that there still are potential unknown factors that still threaten the validity of the results in explaining causal relationship. Also, we could not compare the pregnancy outcomes with the pre-pandemic time due to the lack of access.

Even though there may be support source for pregnant women during pregnancy, our study indicated social support has no moderation impact on the association between perceived risk of COVID-19 and pregnancy outcomes. This probably is due to the nature of the COVID-19 virus that can be transmitted between individuals. This matter may act as a preventive factor to receive any support from the significant others even if they are available to support. Individuals may consider the loved ones’ benefits over their own ones, and this therefore limits the expected positive impact of the social support.

Mindfulness also did not indicate moderating impact on the association between perceived risk of COVID-19 and pregnancy outcomes except for stillbirth. Our results indicated that mindfulness may positively affect the association between perceived risk of COVID-19 and unborn baby’s livability during intrauterine life. Literature lacks to investigate the aforementioned association. However, studies support the positive impact of mindfulness in inducing good outcomes and well-being [55, 56]. This conflict with our results that mindfulness may increase the odd ratio for stillbirth as an adverse pregnancy outcome in response to COVID-19 risk. This may be explainable by less-known nature of COVID-19 infection that lacked the certainty on the possible pregnancy-related consequences and required practices that involves pregnant women’s minds. This matter probably reversed the positive impact of mindfulness as pregnant women may overwhelmed by what needed to be practices, and thus led to increased mental distress, and, in turn, still birth.

Although this study possesses high internal validity, we did not include high-risk pregnancies that could be benefitted more since they are more at risk of developing negative pregnancy outcomes. Another limitation of the present study was non-probability sampling, which should be interpreted with caution.

## Conclusion

Perceived risk of COVID-19 was negatively associated with pregnancy outcomes including birth weight and gestational age at birth. However, social support could not moderate these associations. Mindfulness, on the other hand, moderated the association between perceived risk and stillbirth meaning that by increasing mindfulness, stillbirth may also be increased.

More studies are required to investigate the impact of mindfulness and social support on the association between perceived risk and pregnancy outcomes.

## Data Availability

The datasets generated and analyzed during the current study are not publicly available due to the confidentiality of information, but they can be available through the corresponding author on reasonable request.
